# From despair to hope: tackling Balochistan's suicide crisis with sustainable solutions

**DOI:** 10.1192/bji.2024.8

**Published:** 2024-11

**Authors:** Mehr Muhammad Adeel Riaz, Imrana Imdad, Butool Hisam, Muhammad Zeshan

**Affiliations:** 1 Physician Researcher, Punjab Medical College, Faisalabad, Pakistan; 2 Psychologist, People's Primary Healthcare Initiatives (PPHI), Lasbela, Pakistan; 3 Independent Global Health Policy consultant, Karachi, Pakistan; 4 Clinical Assistant Professor of Psychiatry, Rutgers New Jersey Medical School, Newark, New Jersey, USA

**Keywords:** Suicide, Balochistan, Pakistan, mental health services, public health crisis

## Abstract

Balochistan, Pakistan's largest province, faces a concerning rise in self-harm and suicide, exacerbated by socioeconomic challenges, political instability and events such as climate change and recent suicide bombings. Despite the alarming suicide mortality rate, it remains a neglected public health issue. This perspective paper highlights the need for a comprehensive approach, including suicide prevention initiatives, community-based mental health services, education and research. We also suggest establishing a suicide prevention task force, inclusion of life skills and mental health education in school and undergraduate curricula, conducting research into self-harm, and fostering empathetic media reporting. Additionally, suggestions for sustainable economic change include job creation, development of marketable skills and interest-free microfinance initiatives to empower the community economically.

Balochistan, the largest province of Pakistan in terms of area, has a population of 14.8 million and is currently grappling with an increased trend in self-harm and suicide.^1^ Over the past few decades, the region has faced socioeconomic challenges, political instability, an influx of refugees, and restricted access to developmental infrastructure and human resources, contributing to the state of hopelessness.^2^ The 18th Constitutional Amendment 2011 in Pakistan abolished the Federal Ministry of Health, transferring provincial health reforms to the provincial legislative assembly, but capacity constraints persist in health responsibilities at the provincial level.^2^ Balochistan's mental health system lacks institutional capacity, faces political influence in staffing and suffers from political inefficiency.^2^ This perspective paper aims to illuminate suicide as a public health concern amid additional challenges from natural disasters, climate change and recent suicide bombings^3^ that compound mental health problems and potentially contribute to rising patterns of self-harm and suicide.

## Baluchistan's health system constraints and mental health issues

Overall in Pakistan, and especially in Balochistan, suicidal phenomena remain a relatively neglected and under-researched domain as a public health issue. Official mortality statistics about suicide are conspicuously absent, further impeding the formulation of intervention strategies and suicide prevention initiatives.^4^ Independently collected data suggest 51 deaths by suicide between January and July 2023 in the province.^1^ Presently, the age-standardised suicide mortality rate in Pakistan stands at 9.77 per 100 000 population.^4^ This alarming statistic translates to as many as 15–35 people taking their lives every day, equivalent to approximately one suicide every hour.^5^

Recent events, such as climate change and natural disasters such as the 2022 floods, have induced profound grief, anxiety, loss, fear and sorrow across all four provinces.^6^ The loss of family, food insecurity, homelessness and the absence of mental health services have had a significant impact on the psychological well-being of those residing in flood-affected regions.^6^ Disasters not only affect the quality of life but also impose a substantial burden of mental health challenges on the affected population.^6^ There has been a recent surge in suicide bombings in Balochistan targeting the indigenous population.^3^ Analysing suicide bombing using the frustration–aggression hypothesis sheds light on the psychological dynamics behind the increased incidents in the region, suggesting a link to chronic frustration arising from deprivation and threats to personal (tribal) integrity, encompassing physical risk and self-esteem concerns.^7^

## Legal framework and suicide prevention efforts in Pakistan

Before 2023, any person's admission to hospital following a suicide attempt required mandatory reporting to the police and could lead to their prosecution for a criminal offence under the Pakistan Penal Code 325, with punishment by imprisonment and/or a fine of up to PKR 10 000. The law was repealed in December 2022.^8^ Regrettably, neither a stand-alone nor an integrated short-term strategy or resultant policy for suicide prevention exists at the national or provincial level.^4^ The public, aware of the fragile state of the health sector, increasingly turns to private out-of-pocket services.^2^ Notably, the absence of a dedicated authority or autonomous body tasked with evaluating the compliance of mental health legislation with international human rights norms is a salient shortcoming.^4^

## Determinants and risk factors in suicidal behaviour

Examination of suicidal behaviour, encompassing self-harm, suicidal ideation and completed suicide, has revealed differences in determinants, risk factors and motivations. In Pakistan, interpersonal issues^9^ are prominent factors, along with financial difficulties.^9^ One large study reported that suicide was the most common motive for self-harm (86.1%),^10^ with higher intent reported among those presenting in a community setting compared with those presenting to a hospital. Ingestion of pesticides or toxic chemicals was a frequently reported method of self-harm, particularly among lower socioeconomic strata, youth, females and housewives.^11^ Completed suicide often involved firearms, hanging or organophosphorus poisoning.^11^

## The way forward

Reducing suicide rates requires extensive public health interventions involving socioeconomic and political efforts, and collaboration between scientific and religious communities to positively influence local culture.

### Establishment of a suicide prevention task force and mental health legislation reforms

The provincial government must collaborate with mental health professionals, community leaders (religious and political) and the private sector to develop and implement culturally sensitive and evidence-based suicide prevention initiatives and establish a dedicated suicide prevention task force.^12^ Provincial government could seek technical assistance from private stakeholders such as the Brain and Mind Institute (BMI) at Aga Khan University (AKU), Pakistan.^12^ An autonomous body of mental health professionals and policymakers at the institute (the Self-Harm Registry Committee) is overseeing the development of a self-harm registry at AKU Kenya that will compile, evaluate and ensure compliance with respective nation's mental health legislation.^12^ This must be followed by engaging in dialogue with religious and tribal leaders to positively influence local culture to reduce stigma surrounding mental health problems and move towards making empathetic and evidence-based mental health laws.

### Community-based mental health services

The federal government could collaborate with multilateral organisations^6^ to allocate resources to build the capacity of community-based mental health service providers and interested youth in flood-affected regions, addressing the psychological impact of natural disasters. This process would include collaborating with local non-governmental organisations (NGOs) and mental health professionals to provide counselling, support and psychiatric services to affected communities.^6,11^

### Education and awareness

To improve access to a better life, the provincial government must invest in building schools and educational facilities in rural and marginalised areas, ensuring that every child has access to standardised quality education. School and undergraduate curricula must be enhanced to include life skills, mental health education and career guidance, empowering students with the knowledge and tools they need to navigate challenges effectively.

### Research and data collection on self-harm

Collaborating with multilateral organisations and academic psychiatrists might facilitate research initiatives to collect comprehensive data on self-harm in Balochistan, addressing the current paucity of information. This would encourage national and local academic institutions and mental health researchers to conduct studies into the determinants, risk factors and motivations of self-harm in the region and report to a national suicide registry.^11^

### Empathetic and non-judgemental media reporting

There must be national guidelines emphasising the nuanced and complex nature of suicide and self-harm, steering clear of sensationalism. Federal and provincial governments must engage the Pakistan Electronic Media Regulatory Authority (PEMRA) in empathetic storytelling that brings attention to the profound impact on individuals and families. This may also mean that media outlets must take a proactive stance by including information on available support resources, guiding vulnerable individuals to seek help during times of crisis.

### Strategies for sustainable change

To empower local communities economically, we suggest the following:

- job creation – encourage investment in industries and infrastructure projects to provide employment opportunities for the local population
- skills development – establish vocational training centres to equip individuals with marketable skills and increase their chances of finding employment
- microfinance initiatives – support entrepreneurship by offering microloans and financial literacy programmes to aspiring entrepreneurs, enabling them to start their businesses and generate income.

## Conclusion

Addressing the complex challenges faced by Balochistan necessitates a multi-faceted approach that involves legislative reforms, granular data collection, culturally sensitive community-based services and interdisciplinary collaboration for sustainable economic empowerment of local communities. Implementing these recommendations will contribute to a comprehensive strategy for reducing suicide rates and improving mental health outcomes in the region.

## Data Availability

Data availability is not applicable to this article as no new data were created or analysed in this study.
